# Chanling Gao Attenuates Bone Cancer Pain in Rats by the IKKβ/NF-κB Signaling Pathway

**DOI:** 10.3389/fphar.2020.00525

**Published:** 2020-05-05

**Authors:** Bing Yang, Zhen Zhang, Zhu Yang, Jinghua Ruan, Li Luo, Fengxi Long, Dongxin Tang

**Affiliations:** ^1^Department of Oncology, The First Affiliated Hospital of Guizhou University of Traditional Chinese Medicine, Guiyang, China; ^2^Oncology Team, Guizhou University of Traditional Chinese Medicine, Guiyang, China; ^3^Party Committee Office, Guizhou University of Traditional Chinese Medicine, Guiyang, China; ^4^Department of Oncology, Guihang Guiyang Hospital, Guiyang, China; ^5^Graduate School, Guizhou University of Traditional Chinese Medicine, Guiyang, China

**Keywords:** Chanling Gao, cancer-induced bone pain, IKKβ/NF-κB, Walker 256 breast cancer cells, inflammatory factors

## Abstract

Cancer pain is one of the most common and serious symptoms of cancer patients. At present, the agents used for the prevention or treatment of cancer pain do not act with optimal safety and efficacy. The nuclear factor kappa B (NF-κB) signaling pathway and its downstream inflammatory factors interleukin-6 (IL-6), interleukin-1β (IL-1β), and tumor necrosis factor-α (TNF-α) play an important regulatory role in the developmental process of cancer pain. IKKβ is a key molecule of the IκB (IKK) kinase that propagates cellular responses to inflammation. Previous studies have shown that phosphorylation and degradation of the IκBα protein promotes the activation of NF-κB and the expression of TNF-α, IL-1β, and IL-6, participating in the formation and development of cancer pain. Chanling Gao (CLG) is a compound preparation of traditional Chinese medicine. It contains specific functions, namely nourishing Yin, activating blood circulation and relieving pain and dysfunction syndrome. It is used in the treatment of a variety of pain disorders including cancer-induced bone pain (CIBP), which has a certain relief effect. However, its mechanism of action still remains unclear. In the present study, a rat model of tibia CIBP was successfully established using the Walker 256 breast cancer cell line. The IKKβ/NF-κB signaling pathway and its related factors TNF-α, IL-1β, and IL-6 were used as the entry points to explore the effect of CLG on CIBP and their possible mechanisms of action. The results indicated that CLG improved the body mass of the CIBP rat model and increased the pain threshold in rats. CLG significantly inhibited the degradation of IκBα and the levels of p-IκBα, p-IKKβ, and p-p65 NF-κB proteins in the spinal cord of CIBP rats, inhibiting the contents of TNF-α, IL-1β, and IL-6. Therefore, we conclude that the analgesic effect of CLG in this rat model of CIBP may be related to the inhibition of the IKKβ/NF-κB signaling pathway and the reduction of synthesis and release of TNF-α, IL-1β, and IL-6.

## Introduction

According to the World Health Organization, 30 to 50% of all cancer patients have symptoms of pain. Approximately 15% of all patients with early stage cancer and approximately 60% to 90% of patients in the advanced stage of cancer have clinical manifestations of pain. From the perspective of pain, 40 to 50% of patients have moderate-to-severe pain, and 25% to 30% of patients experience very severe pain (Caraceni and Shkodra, 2019). The diagnosis and treatment of cancer pain is still regarded a very serious complication in clinical practice. At present, cancer pain is treated based on the principle of the “three-step analgesic therapy”; however, due to drug resistance, side effects of drugs, psychological factors, and individual differences, the effects of clinical treatment are not optimistic and seriously affect the quality of life of the patients (Sun and Gu, 2002; Yao et al., 2019). Tramadol hydrochloride is a centrally acting opioid analgesic that is often used clinically for the treatment of various acute and chronic pains, such as cancer pain, fractures, or postoperative pain due to its rapid and complete absorption and high bioavailability (Kim et al., 2019; Musich et al., 2020). However, certain adverse reactions may occur following administration of this drug (Kim et al., 2019; Musich et al., 2020). Previous studies have found that the analgesic mechanism of tramadol hydrochloride may be associated with the activation of the NF-κB signaling pathway (Wojciech and , 2009; Zhou et al., 2015). Chinese medicine is one of the components of multi-disciplinary comprehensive treatment of cancer, which exerts a certain relief on adjuvant treatment of cancer pain, significantly improving the patient symptoms and as a result the quality of life in patients (Wang et al., 2019). This regimen is associated with lesser adverse reactions (Wang et al., 2019). Therefore, it is of great clinical significance to explore effective drugs for preventing and treating cancer pain with the aid of traditional Chinese medicine.

Cancer-induced bone pain (CIBP) is the most common type of cancer pain and is the main cause of poor quality of life in patients suffering from cancer. Nuclear factor kappa B (NF-κB) is an important transcription factor in the body that plays a significant role in the regulation of immune and inflammatory responses. Similarly, IKKβ is also a key molecule of the IκB (IKK) kinase that propagates cellular responses to inflammation. It has been reported that the IKKβ/NF-κB pathway is involved in the formation and developmental processes of cancer pain, inflammatory pain, mechanical hyperalgesia, and thermal hyperalgesia (Wang et al., 2018; Tian et al., 2019). The IκB kinase is activated and phosphorylated under the stimulation of inflammatory factors, followed by ubiquitination and degradation. This process in turn promotes the entry of NF-κB into the nucleus and initiates the expression of its corresponding target genes, inducing several target proteins. The synthesis of these proteins exhibits certain biological effects (Jing et al., 2015; Oguiza et al., 2015). The inflammatory response is closely associated with the development of cancer pain. Previous studies have shown that the proinflammatory cytokines, such as interleukin-6 (IL-6), interleukin-1β (IL-1β), and tumor necrosis factor-α (TNF-α) play an important role in the development and progression of neuralgia in the central nervous system, eventually leading to central sensitization and formation of persistent pain (Nadeau et al., 2011; Doong et al., 2015; Lu et al., 2015; Hung et al., 2017). Activation of NF-κB in the spinal cord tissues of rats with neuropathic pain and inflammatory pain indicates close association with the production and maintenance of pain, while inhibition of NF-κB activation significantly alleviates pain and inflammation in rats (Fu et al., 2010). According to previous studies, the expression of NF-κB is significantly increased in the spinal cord of CIBP rats, suggesting that the occurrence of CIBP is related to the activation of the NF-κB pathway (Zhou et al., 2015). Stimulation of pro-inflammatory cytokines activates TNF-α, IL-1β, and IL-6 and NF-κB. The activated NF-κB regulates the expression levels of IL-1β, IL-6, and TNF-α and the two feedback each other to promote the occurrence and development of pathological neuralgia (St-Jacques and Ma, 2011; Hoesel and Schmid, 2013; Chen et al., 2019). In summary, the IKKβ/NF-κB signaling pathway and the pro-inflammatory cytokines TNF-α, IL-1β, and IL-6 play important regulatory roles in the development of cancer pain.

**Figure 1:**
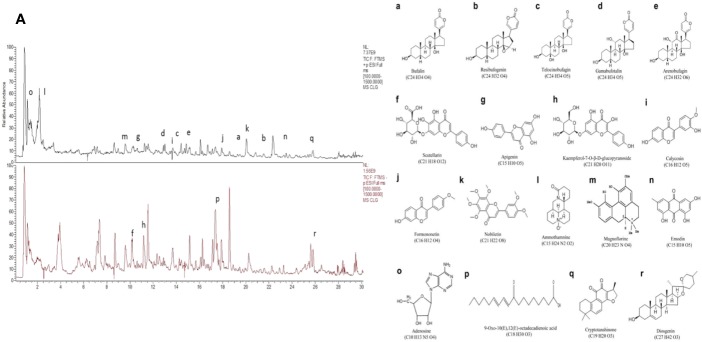
The identification of the active compounds of CLG as determined by HPLC. **(A)**. HPLC of CLG. **a**. Bufalin. **b**. Resibufogenin. **c**. Telocinobufagin. **d**. Gamabufotalin. **e**. Arenobufagin. **f**. Scutellarin. **g**. Apigenin. **h**. Kaempferol-7-O-β-d-glucopyranoside. **i**. Calycosin. **j**. Formononetin. **k**. Nobiletin. **l**. Ammothamnine. **m**. Magnoflorine. **n**. Emodin. **o**. Adenosine. **p**. 9-Oxo-10(E),12(E)-octadecadienoic acid. **q**. Cryptotanshinone. **r**. Diosgenin.

Chanling Gao (CLG, Patent No.: 201410626601.3) is a Chinese traditional medicine used in Liu Shangyi's clinical experience for more than 40 years. It is mainly composed of skin of toad (Bufo gargarizans Cantor) and other traditional Chinese medicinal components and is used to treat benign and malignant masses, causing a relief effect on cancer pain in various advanced tumors (Huang et al., 2017). The application of CLG in the First Affiliated Hospital of Guizhou University of Traditional Chinese Medicine since 2010 enabled the treatment of nearly 10,000 cancer patients. Modern pharmacological research suggests that the active constituents of the main components of CLG, such as Chan Pi (*Toad Skin*), Wei Ling Xian (*Clematis chinensis*), Ge Gen (*Radix Puerariae*), Chuan Xiong (*Ligusticum chuanxiong Hort*), and E Zhu(*Curcuma aeruginosa Roxb*) exhibit anti-tumor effects that reduce the side effects of chemotherapy and improve immunity (Cao et al., 2015; Lee et al., 2015; Zhang et al., 2018; Hao et al., 2019; Zhan et al., 2020). Our previous study demonstrated that the clinical application of CLG (Huang et al., 2016; Yang et al., 2016; Yang et al., 2019) exhibited a certain relief effect in patients with cancer pain and a significant effect on a colorectal cancer nude mouse model. The HPLC (**Supplementary Material**: **Figure 1**, **Tables 1** and **2**) analysis of CLG indicated (**Figure 1A**) that this extract mainly contained bufogenins [bufalin (0.47 μg/ml), resibufogenin (0.49 μg/ml), telocinobufagin (0.32 μg/ml), gamabufotalin (0.28 μg/ml), arenobufagin (0.36 μg/ml)] (**Figures 1a–e**), flavonoids [scutellarin (0.26 μg/ml), apigenin (0.65 μg/ml), kaempferol-7-O-β-d-glucopyranoside (0.41 μg/ml), calycosin (1.37 μg/ml), formononetin (0.44 μg/ml), and nobiletin (0.72 μg/ml)] (**Figures 1f–k**), alkaloids [ammothamnine (0.36 μg/ml) and magnoflorine (0.98 μg/ml)] (**Figures 1l, m**) as well as other ingredients, such as emodin (0.39 μg/ml), adenosine (0.56 μg/ml), 9-Oxo-10(E),12(E)-octadecadienoic acid (0.18 μg/ml), cryptotanshinone (0.55 μg/ml), and diosgenin (0.58 μg/ml) (**Figures 1n–r**). However, the specific role of CLG in cancer pain and its mechanism remain unclear. Inflammatory factors are important components for the occurrence and development of cancer pain and therefore, inhibition of inflammatory factors plays an important role in the prevention and treatment of cancer pain.

## Materials and Methods

### Animal Model

The Walker 256 breast carcinoma cell line was purchased from the ATCC cell bank (ATCC Number: XB-2496, Shanghai, China). The Walker 256 cells that were collected in the logarithmic growth phase were inoculated into the abdominal cavity of 4-week-old female Wistar rats weighing 60 to 80 g that were purchased from Hunan Changsha Tianqin Co., Ltd. [experimental animal license number SCXK (Liao) 2015-0001], after 10 days, the ascites was collected waiting to use. A rat model of bone cancer pain established by following the method of Medhurst et al. (2002). First, the rats were anesthetized with 10% chloral hydrate (3 g/kg) by intraperitoneal injection [weighing 160–180 g, 5-week-old male rats, purchased from Hunan Changsha Tianqin Co., Ltd., experimental animal license No. SCXK (Liao) 2015]. After the rats were anesthetized, they were fixed in the supine position on the operating table. When the cornea reflex and the clamp response disappeared, the tibia of the right hind limb of the rat was shaved and disinfected. Second, cut the middle and upper sections of the tibia to expose the tibial bone surface, and then use a 1-mm diameter stainless steel drill to make holes, but prevent the tibia from being punctured. Third, when the tibial foramen was unobstructed, 10 μl (3×10^4^/μl) of ascites containing Walker256 cells was injected into the bone marrow cavity using a micro-syringe, and then the drilling site was quickly closed with bone wax, and only 10 μl of the normal saline was injected into the rats in the sham operation group (Sham). Fourth, disinfect the wound and then perform layered sutures, and the entire operation strictly adheres to the aseptic operation. The rats were tested on days 7, 14, and 21 of modeling using hot and cold plates (DB026, Beijing Zhimoo Duobao Biotechnology Co., Ltd., Beijing, China) and von frey (IITC-2393, USA) electronic pain meter. These rats were housed under specific-pathogen free (SPF) conditions at a temperature of 24 ± 0.5°C and in a relative humidity of 50% to 60%. All experimental procedures were approved by the Medical Ethics Committee of Guizhou University of Traditional Chinese Medicine.

### Groups and Administration

Following successful modeling, the model rats were randomly divided into the model control group, the Changling Gao high (CLGH), the Chanling Gao middle (CLGM), the Chanling Gao low (CLGL), and the tramadol hydrochloride (QMD) groups.

The dose of CLGM rats administered was the equivalent dose of CLG used clinically in adults. CLG was provided by the First Affiliated Hospital of Guizhou University of Traditional Chinese Medicine (100 g/bottle, brown paste, batch number: 20180827). The CLGH group was administered with 8 g (raw drug)/Kg (body mass)/d (800 g/l) aqueous solution (2 ml gavage), the CLGM group with 4 g/Kg/d (400 g/l) aqueous solution (2 ml gavage) and CLGL group with 2 g/Kg/d (200 g/l) of an aqueous solution of 2 ml. The Sham group and the model control group were administered intragastrically saline (2 ml/d) for 7 days. The QMD group (purchased in Shanghai Xudong Haipu Pharmaceutical Co., Ltd., specification: 0.025 g/tablet, 25 mg×10 pieces/box, batch number: FA170304) rats were administered 2 ml 0.02 g/kg (0.02 g/l) aqueous solution for 7 days.

### Effect of CLG on Model Rats

The general condition of the rats was observed daily, including their diet, stool, spirit, dull hair, and sensitivity to irritation. The body weight of the rats was measured every 7 days, and the body mass growth curve of the nude mice was measured to observe the effects of CLG. Concomitantly, the paw withdrawal latency to heat stimulation [PWL: The rats were placed in a glass plate chamber, and the palms of the hind toes were stimulated with a hot and cold plate in a quiet awake state, The time (s) from the start of the stimulus to the occurrence of footlift avoidance is PWL. Repeat the measurement three times for each rat, each interval is at least 3 min, and the PWL value is an average of 3 times.] and paw withdrawal threshold to mechanical stimulation [PWT: Von Frey filament was used to stimulate the depression of the plantar skin of the hind limbs, the filament is bent half and lasts for no less than 5 s. When rats lift or lick their feet, it is a positive reaction, otherwise it is a negative reaction, and the positive response is recorded in grams (g).] of the rats were measured every 7 days during the administration of hot and cold plates (DB026, Beijing Zhimoduo Biotech Co., Ltd., Beijing, China) and von frey (IITC-2393, USA).

### X-ray Film

The parts of each group on days 7, 14, and 21 following modeling were filmed by X-rays to observe the bone destruction caused by the tumor of the affected limb.

### Hematoxylin and Eosin (H&E)

Following the last administration, the rats were sacrificed the next day, and the tibia of the model site was obtained. The tibia was subsequently fixed with 10% formalin, decalcified, dehydrated, soaked to transparency, dipped in wax, embedded, and converted into 5 m thick slices. The specimens were subsequently stained with H&E to observe bone destruction.

### Immunohistochemistry

Paraffin sections of spinal cord tissues were 5 μm thick and stained with the SP kit (purchased from Beijing Zhongshan Jinqiao Biological Co., Ltd., Beijing, China) according to the routine immunohistochemical procedure. The dilution concentrations of the primary antibodies were the following: TNF-α, 1: 200, (ab6671, ABCAM); IL-1β, 1:200 (ab9722, ABCAM); and IL-6, 1:200 (ab9324, ABCAM). Subsequently, the sections underwent DAB color development and were photographed under an inverted microscope. Each immunohistochemical section was randomly observed under five fields of view and the number of positive cells per unit area was calculated. According to the percentage of positive cells, the scoring was divided into the four following levels: negative expression (< 5%, −); weak positive expression (5–25%, +); positive expression (25–75%, ++); strong positive expression (75–100%, +++) (Tang et al., 2019).

### Western Blot Analysis

A total of 100 mg of spinal cord tissue was obtained from each group, sonicated on ice, and centrifuged at 13,500 rpm for 30 min at 4°C. The supernatant protein solution was extracted, quantified, diluted with 5 X loading buffer in order to denature the protein. Subsequently, 50 μg protein was loaded on 10% SDS polyacrylamide gels for immunoblotting. The primary antibodies were diluted with 3% skimmed milk as follows: IκBα (1:1,000, E130, ab32518, ABCAM), p-IκBα (1:1,000, SC-8059, Santa Cruz), IKKβ (1:1,000, EPR6043, ab124957, ABCAM), p-IKKβ (1:1,000, 2697S, CST), p65 NF-κB (1:1,000, 8242S, CST), p-p65 NF-κB (1:1,000, 3033S, CST). The antibodies were incubated with the membranes overnight at 4°C. The secondary antibodies of the corresponding sources were used separately. The gray values of the protein bands were analyzed by the Bandscan software (Tang et al., 2019).

### ELISA

Total blood from the rats was collected at the end of the experiment, centrifuged at 3,000 rpm for 15 min, and subsequently, the supernatant was aspirated to determine TNF-α and IL-1β levels by ELISA (ERC102a.96, ERC007.96, ERC003.96, Shenzhen Xinbosheng). The content of IL-6 was also determined.

### Statistical Analysis

The data obtained were expressed as mean ± standard error (mean ± SEM) and were statistically analyzed by the GraphPad Prism 5 statistical software (GraphPad software Inc, USA). The one-way analysis of variance (ANOVA) method was used to determine significant differences. The comparison between the groups was performed by the Bonferroni correction. A *P* value lower than 0.05 (*P < 0.05) was considered for significant differences.

## Results

### The CIBP Rat Model Was Successfully Established

**Figure 2:**
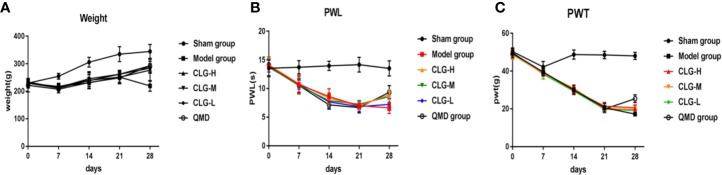
Effect of CLG on body mass, the paw withdrawal latency to heat stimulation (PWL) and the paw withdrawal threshold to mechanical stimulation (PWT) in the CIBP rat model. **(A)** Growth curve of body weight of rats in each group (n=6). **(B)** The PWL of rats in each group (n = 6). **(C)** The PWT of rats in each group (n=6). In the model group, the PWL and PWT were decreased on the 7th, 14th, and 21st day following surgery, and no significant changes were noted in the sham group. On day 21 of administration, and 7 days following administration, the PWL and PWT were increased in the CLGH, CLGM, CLGL, and QMD groups and the model control group indicated a continuous decrease, whereas the sham group indicated no significant change.

**Figure 3:**
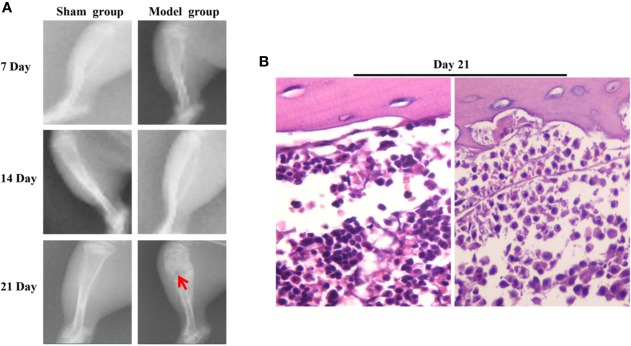
The results of X-ray and H&E of tibia of CIBP rats. **(A)** On the 7, 14, 21st day the X-ray results of tibia in the sham and model groups indicated that the arrow pointed to the site of tibia destruction. It was found that the tibial structure of the model group was significantly damaged, whereas the local bone density was uneven, with loss of cortical bone defect, swelling of surrounding muscles and soft tissues. However, in the Sham group, no abnormalities were noted in the tibia, the bone density was uniform, and the cortical bone was continuous. No deletion was present. **(B)** H&E results of tibia in the sham group and the model group (400×). The results indicated that the bone marrow cavity of the model group was filled with a large number of tumor cells, the trabecular bone was destroyed, the bone structure was seriously lost and the surrounding muscle and soft tissues were destroyed by the tumor cells. Various normal bone marrow cells were observed in the bone marrow cavity of the sham group and the trabecular and cortical bones were intact without any obvious abnormalities.

In the model group, the PWL and PWT were decreased on days 7, 14, and 21 following surgery. No significant changes were noted in the PWL in the sham group, while the PWT was decreased in the first 7 days following surgery. Subsequently it was increased on days 7 to 14 and decreased slowly after the 14th day (**Figures 2B, C**). The X-ray films of the rat tibia were obtained on days 7, 14, and 21 of the model, and it was found that on the 21st day, the tibial structure of the model group was significantly damaged and the local bone density was uneven, with loss of bone structure, cortical bone defect, and swelling of surrounding muscles and soft tissues (**Figure 3A**). However, in the sham group, no abnormalities were present in the tibia and the bone density was uniform (**Figure 3A**). The cortical bone was continuous in the absence of bone deletion (**Figure 3A**). H&E analysis indicated that the bone marrow cavity of the model group was filled with a large number of tumor cells, whereas the trabecular bone was destroyed, the bone structure was seriously depleted and the surrounding muscle and soft tissues were destroyed by the tumor cells (**Figure 3B**). Various normal bone marrow cells were observed in the bone marrow cavity of the sham group, and the trabecular bone and cortical bone were intact without any apparent abnormalities (**Figure 3B**). These results suggested that the CIBP model was successfully established (Medhurst et al., 2002; Khasabova et al., 2011).

### CLG Can Improve the General Condition of Model Rats

The rats in the sham-operated group were fed during the whole experiment, and they exhibited normal stools, shiny hair, and were in good shape. These animals were sensitive to irritation and had no limp. Following 7 days of model establishment, the rats in the model group indicated decreased food intake, decreased activity, persistent wilting, dull hair, claudication, and progressive aggravation. Following 3 to 7 days of administration of CLG and tramadol, the food intake of the rats in each group was increased, and they showed significant improvement in their vital signs. Moreover, their activity was increased, the gloss of the hair showed improvement, and the lameness was also improved. Following saline administration in the rats of the model control group, their food intake and tiredness were reduced, whereas their vital signs were decreased. The hair of the animals was dull. No significant changes in the sham operation group were noted. These results suggested that CLG could improve the general vital signs and health of the CIBP model rats.

### CLG Can Improve the Body Weight of Model Rats

The results of the body mass growth curve revealed that the body mass of the sham group rats was gradually increased on days 7, 14, and 21 following modeling, while the rats in the model group exhibited weight loss (**Figure 2A**). The body mass of the rats following 7 days of intervention with CLG, QMD, and saline indicated a continuous increase in the sham group. The groups of CLGH, CLGM, CLGL, and QMD exhibited a slow increase and the model control group indicated a continuous decrease (**Figure 2A**). This suggested that CLG could improve the body mass of CIBP model rats.

### CLG Increases PWL and PWT of Model Rats

In the model group, the parameters PWL and PWT were decreased on 7, 14, and 21 days following surgery, and no significant changes were noted in the sham group (**Figures 2B, C**). On day 21 of administration and following 7 days of administration, the parameters PWL and PWT were increased in the CLGH, CLGM, CLGL, and QMD groups, whereas the model control group indicated a continuous decrease and the sham group revealed no significant change (**Figures 2B, C**). This suggested that CLG could improve the pain of CIBP rats.

### CLG Significantly Inhibits IκBα Protein Degradation and p-IκBα, p-IKKβ, p-p65 NF-κB Protein Levels in the Spinal Dorsal Horn

**Figure 4:**
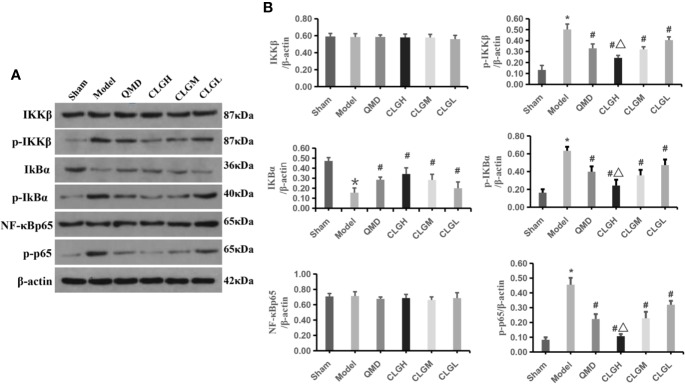
The regulating effect of CLG on related proteins. **(A)** Inhibitory effects of CLG on the expression of IκBα (p-IκBα), IKKβ (p-IKKβ) and NF-κB (p-p65 NF-κB) in the transplanted tumor as determined by western blot analysis. **(B)** Semi quantification of the protein levels of IκBα (p-IκBα), IKKβ (p-IKKβ) and NF-κB (p-p65 NF-κB) in the sham, model and in the QMD, CLGH, CLGM, and CLGL groups. The data are mean ± SEM (n = 6). *P < 0.05 vs. sham, ^#^*P* < 0.05 vs. Model, ^△^P < 0.05 vs. CLGL and CLGM.

Previous studies have shown that tramadol hydrochloride can significantly improve cancer pain (Wojciech and , 2009), and the occurrence of cancer pain may be related to the activation of the NF-κB signaling pathway (Zhou et al., 2015). Western blotting demonstrated that the expression levels of the p-IκBα, p-IKKβ, and p-p65NF-κB proteins were significantly increased compared with those of the sham group (*P* < 0.05). IκBα protein levels were significantly decreased (*P* < 0.05), whereas IKKβ and NF-κBp65 protein levels exhibited no significant differences in the spinal dorsal horn of the model control group (*P* > 0.05) (**Figures 4A, B**). The levels of p-IκBα, p-IKKβ, and p-p65NF-κB in the spinal dorsal horn of the CLGH, CLGM, CLGL, and QMD group rats were significantly lowered (*P* < 0.05) and the IκBα protein levels were significantly increased compared with those of the model control group (*P* < 0.05), (**Figures 4A, B**). The levels of p-IκBα, p-IKKβ, and p-p65NF-κB proteins in the spinal dorsal horn of the CLGH group rats were significantly decreased (*P* < 0.05), while IκBα protein levels were significantly increased compared with those of the CLGM and CLGL groups (*P* < 0.05) (**Figures 4A, B**). This suggested that CLG could significantly inhibit the degradation of the IκBα protein and reduce the levels of the p-IκBα, p-IKKβ, and p-p65 NF-κB proteins in spinal cord tissues at a dose-dependent relationship.

### CLG Significantly Inhibits TNF-α, IL-1β, and IL-6 Protein Levels in Spinal Cord and Serum Samples

**Figure 5:**
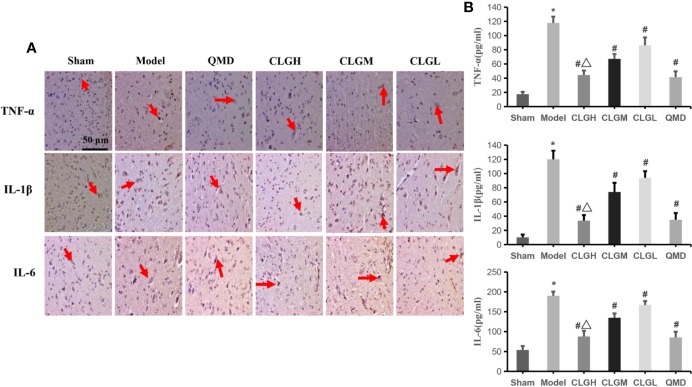
Inhibitory effects of CLG on the inflammatory factors in the rat models of bone cancer pain. **(A)** Immunohistochemical results of TNF-α, IL-1β, and IL-6 proteins in rat spinal cord (n = 3). The arrows indicate the positive expression. **(B)** Elisa results of TNF-α, IL-1β, and IL-6 proteins in rat serum (n = 6). *P < 0.05 vs. sham, ^#^*P <* 0.05 vs. Model, ^Δ^*P <* 0.05 vs. CLGL and CLGM.

The immunohistochemical results indicated that TNF-α, IL-1β, and IL-6 proteins exhibited a strong positive expression (+++) in the model control group, weak positive expression (+) in the CLGH and QMD groups and positive expression (++) in the CLGM and CLGL groups (**Figure 5A**). ELISA results indicated that the levels of TNF-α, IL-1β, and IL-6 in the serum of the model control group were significantly higher than those in the sham group (*P* < 0.05) (**Figure 5B**). The serum levels of TNF-α, IL-1β, and IL-6 in the CLGH, CLGM, CLGL, and QMD groups were significantly lower compared with those of the model control group (*P* < 0.05) (**Figure 5B**). The TNF-α, IL-1β, and IL-6 protein levels were significantly lower in the CLGH group compared with those of the CLGM and CLGL groups (*P* < 0.05) (**Figure 5B**). These results suggested that CLG could significantly inhibit the synthesis and release of the cytokines, TNF-α, IL-1β, and IL-6 in the spinal cord as well as in the serum in a dose-dependent manner.

## Discussion

The present study demonstrated that CLG could improve the body mass of the tibia in a CIBP rat model established by the Walker 256 breast cancer cell line and improve the thermal pain threshold as well as the mechanical pain threshold of the model rats, thereby alleviating cancer pain. CLG significantly inhibited the degradation of the IκBα protein and the phosphorylation of IκBα, IKKβ, and p65NF-κB proteins in the spinal cord of CIBP rats, which in turn inhibited the contents of TNF-α, IL-1β, and IL-6. Therefore, we suggest that the analgesic effect of CLG on the CIBP rat model may be related to the regulation of the IKKβ/NF-κB signaling pathway and the reduction in the synthesis and release of TNF-α, IL-1β, and IL-6.

CLG is a traditional Chinese medicinal compound based on dried toad skin (Bufo gargarizans Cantor). The HPLC analysis of CLG indicated that CLG mainly contained bufogenins, flavonoids, alkaloids, and other ingredients. Previous studies have shown that bufogenins (Ba et al., 2018; Hao et al., 2019), flavonoids (Kono et al., 2014; Gui et al., 2018) and alkaloids (Gutridge et al., 2019) can significantly reduce the occurrence of cancerous pain. The associated mechanism of action may be related to the reduction in the levels of the proinflammatory cytokines TNF-α, IL-1β, and IL-6 by regulating the NF-κB pathway.

CIBP is the most common type of cancer pain. It causes severe pain and is more commonly observed in a variety of primary bone cancers. Also it is caused by metastasis of other malignant tumors, such as bone metastasis of breast cancer, lung cancer, and prostate cancer. The pathogenesis of cancer pain remains complex and unclear. The features of cancer pain include inflammatory pain and neuropathic pain. The mechanisms involved in this process include cancer cell proliferation and infiltration, central and peripheral nerve facilitation, and imbalance of inflammation and immune responses (Diaconu et al., 2015; Fagundes et al., 2015). It has been reported that NF-κB plays an important role in the regulation of inflammatory response *in vivo*. Previous studies have revealed that the NF-κB signaling pathway is involved in the regulation of the expression of various inflammatory mediators present in pathological neuralgia, in aggravation of the inflammatory response and in the induction of pain (Wang et al., 2018; Tian et al., 2019). Previous studies have reported the activation of NF-κB in the spinal cord of CIBP rats. Intrathecal injection of NF-κB antagonists alleviates hyperalgesia induced by CIBP in rats. Activation of NF-κB induces or promotes the formation of tibia in rats with CIBP (Song et al., 2016; Wu et al., 2018). As the N-terminus of NF-κB has a Rel homology region of approximately 300 amino acid residues, it is collectively referred to as the NF-κB/Rel protein family, which mainly includes the Rel A (p65), c-Rel, Rel B, NF-κB1 (P50), and NF-κB2 (P52) transcription factors. According to previous studies, the expression of phosphorylated NF-κB-p65 and its nuclear translocation are increased during pain (Gupta et al., 2011). IKK includes the three subunits IKKα, IKKβ, and IKKγ, of which IKKβ plays a particularly important role in the activation of the classical NF-κB signaling pathway (Prescott and Cook, 2018). In a large number of cell types, IKKβ is regarded a key IκB kinase, and phosphorylation of IκBα by phosphatases is a necessary condition for NF-κB activation. However, this step alone is not sufficient for NF-κB activation (Gao et al., 2014). IKKβ is another important factor that plays a vital role in promoting the activation of NF-κB by stimulating phosphorylation and degradation of the IκBα protein. The present study demonstrated that CLG exhibited increasing effects of thermal pain and mechanical pain thresholds in model rats, thereby alleviating cancer pain. Western blot analysis indicated that CLG significantly inhibited p-IκBα, p-IKKβ, and p-p65NF-κB protein levels and upregulated the expression of IκBα protein in the spinal cord of CIBP rats. Therefore, the data suggested that the analgesic effect of CLG on CIBP rats might be associated with the inhibition of phosphorylation of the IKKβ protein by CLG, thereby inhibiting the activation of IκBα and consequently the activation of NF-κB.

Cytokines are small-molecular weight proteins with a wide range of biological activities. They are synthesized and secreted by immune cells and certain non-immune cells and possess a wide-ranging role in regulating cellular functions and the occurrence of pain. The tumor tissues can release a range of cytokines, chemical factors, and growth factors. For example, most of the receptors, such as TNF-α, IL-6, interleukin-1 (IL-1), prostaglandin (PGE), and endothelin (ET) can be expressed on primary sensory neurons (Tang et al., 2016; Khodorova et al., 2018). It has been shown that IL-6, IL-1β, and TNF-α play an important role in the pathogenesis and development of pathological neuralgia, eventually leading to central sensitization and formation of persistent pain, resulting in reduced quality of life in patients (Hung et al., 2017). IL-1β and IL-6 are considered important regulatory mediators in the neuro-endocrine-immune system. External noxious stimuli increases the expression of IL-1β and IL-6, which in turn is directly associated with the degree of acute inflammatory response and the degree of pain (Fang et al., 2015). TNF-α increases the synthesis of prostaglandin E2, bradykinin, and substance P through the cyclooxygenase-2 pathway, which in turn leads to hyperalgesia and increased neuronal excitability (de Araujo et al., 2015). Previous studies also found that activated NF-κB regulates the expression of genes such as IL-1β, IL-6, and TNF-α and promotes the occurrence and development of pathological neuralgia (Zhang et al., 2015; Su et al., 2017). IKKβ acts as a key molecule of IκB kinase and phosphorylation and degradation of the IκBα protein promotes the activation of NF-κB (Jing et al., 2015; Oguiza et al., 2015). Cancer pain is closely associated with IKKβ/NF-κB, IL-1β, IL-6, and TNF-α. Inhibition of IKKβ enzyme activity and IκBα protein phosphorylation and degradation has an important inhibitory effect on NF-κB protein activation. It further causes an important inhibitory effect on the expression of the downstream inflammatory factors TNF-α, IL-1β, and IL-6. In the present study, CLG was shown to improve pain in the CIBP rat model. Western blot analysis indicated that CLG inhibited phosphorylation of IκBα, IKKβ, and p65NF-κB proteins in the spinal cord of the CIBP rat model, whereas it upregulated the levels of the IκBα protein (**Figures 4A, B**). Immunohistochemical analysis demonstrated that TNF-α, IL-1β, and IL-6 exhibited a strong positive expression in the model control group, a weak positive expression in the QMD and CLGH groups and a positive expression in the CLGM and CLGL groups (**Figure 5A**). Furthermore, the ELISA results indicated that the levels of TNF-α, IL-1β, and IL-6 in the serum of the model control group were significantly higher than those in the sham group (*P* < 0.05) (**Figure 5B**). The serum levels of TNF-α, IL-1β, and IL-6 in the CLGH, CLGM, CLGL, and QMD groups were significantly lower compared with those of the model control group (*P* < 0.05) (**Figure 5B**). These results suggested that CLG improved the overall vital signs, health, and pain reduction in the CIBP rat model, while inhibiting the degradation of the IκBα protein and the phosphorylation of the IκBα, IKKβ, and p65NF-κB proteins in the spinal cord of rats with CLG inhibition. The role of TNF-α, IL-1β, and IL-6 proteins was inter-related.

In conclusion, the results of the present study indicated that CLG could improve the general quality of life and body mass in a rat CIBP model, while increasing the thermal and mechanical pain thresholds in model rats, thereby alleviating cancer pain. Its mechanism may be related to the inhibition of the IKKβ/NF-κB signaling pathway, which was involved in the reduction of the synthesis of the pathway-associated proteins and the release of the inflammatory factors TNF-α, IL-1β, and IL-6.

The present study can be further improved. A shorter dosage cycle can be used and the question of whether CLG improves the bone destruction of CIBP rats can be addressed. Moreover, the survival rate of CLG in the CIBP rat model and the analgesic effect of CLG combined with QMD in the CIBP rat model can be further explored. Although the results of the present study suggested that the analgesic effect of CLG on CIBP rats might be related to the inhibition of its related biological factors, their specific targets and their association were not clarified. We hope to continue to improve these limitations in our subsequent studies.

## Data Availability Statement

The data sets generated for this study are available on request to the corresponding author.

## Ethics Statement

The animal study was reviewed and approved by The Medical Ethics Committee of Guizhou University of Traditional Chinese Medicine.

## Author Contributions

BY, ZZ, ZY, JR, FL, and DT designed the study. ZZ, FL, LL, and DT generated the data. ZZ, BY, FL, and DT analyzed the data. BY and DT prepared manuscript draft. All authors approved the final manuscript.

## Funding

This work was financially supported by the National Natural Science Foundation of China (No. 81760814, No. 81860819, No. 81960818), Guizhou Traditional Chinese Medicine Tumor Inheritance and Science and Technology Innovation Talent Base (No. Deaf leader-[2018] No. 3), Yang Zhu, Guizhou Province, “Traditional Chinese Medicine Oncology” Graduate Tutor Studio (No. Teaching and research GZS-[2016]08), Guizhou high-level innovative talent training plan (100 levels) (No. Yankehe Talents (2016) No. 4032), TCM graduate school workstation (No. Teaching and research JYSZ-[2014]018), Preclinical study of Chinese medicine master Liu Shangyi's experience Fang Chanling Gao (No. Building branch [2019]9-2-1), Establishment of Zebrafish Colorectal Cancer Model and Evaluation of Chanling Gao (No. Building branch [2019]9-2-35, Guizhou Province Traditional Chinese Medicine Oncology Inheritance and Scientific and Technological Innovation Talent Team (Qian Kehe Platform Talents [2020] 5013), based on HIF-1α/TNF-α-mediated EMT effect to explore the mechanism of Chanling Ointment combined with capecitabine on liver metastasis of colorectal cancer with enhanced attenuating effect (Qianke Foundation [2020] 1Y368).

## Conflict of Interest

The authors declare that the research was conducted in the absence of any commercial or financial relationships that could be construed as a potential conflict of interest.
