# Supplementary figures and images for: Chanling Gao Attenuates Bone Cancer Pain in Rats by the IKKβ/NF-κB Signaling Pathway
Source: Front Pharmacol. 2020 May 5;11:525. doi: 10.3389/fphar.2020.00525 (PMC7214814; doi:10.3389/fphar.2020.00525)

RT: 0.00 - 50.01

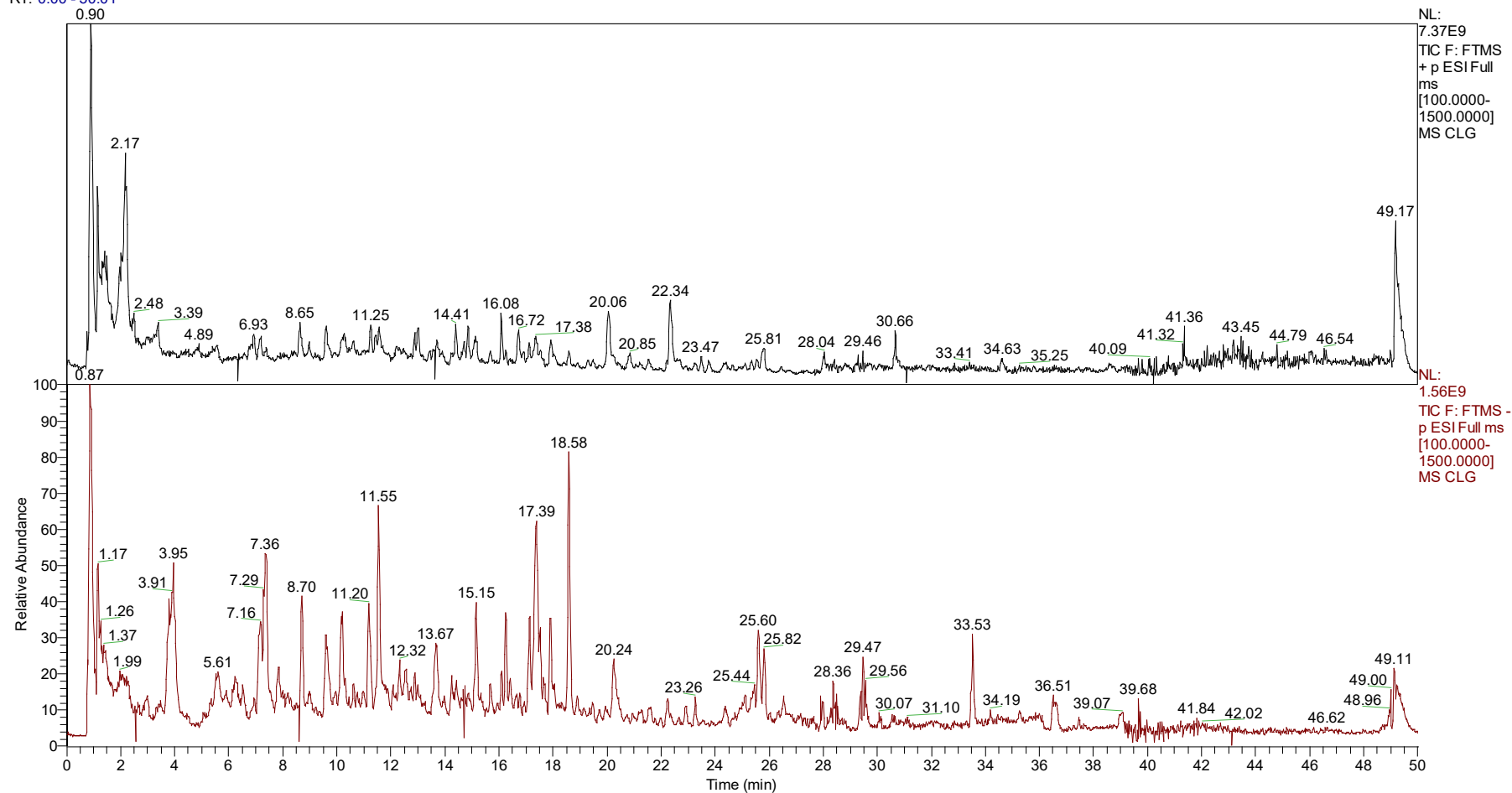

NL:  
7.37E9  
TIC F: FTMS  
+ p ESI Full  
ms  
[100.0000-  
1500.0000]  
MS CLG

NL:  
1.56E9  
TIC F: FTMS -  
p ESI Full ms  
[100.0000-  
1500.0000]  
MS CLG

CLG HPLC mass spectrum

Supplement: Supplementary file 1 [file Image_1.pdf]
