# Supplementary material for: Chanling Gao Attenuates Bone Cancer Pain in Rats by the IKKβ/NF-κB Signaling Pathway
Source: Front Pharmacol. 2020 May 5;11:525. doi: 10.3389/fphar.2020.00525 (PMC7214814; doi:10.3389/fphar.2020.00525)
Supplement: Supplementary file 2 [file Table_2.doc]

| project | description | manufacturer |
| --- | --- | --- |
| Liquid phase system | Dionex Ultimate 3000 RSLC (HPG) | Thermo Fisher Scientific |
| Mass spectrometry system | Thermo Scientific Q Exactive Focus | Thermo Fisher Scientific |
| source of ion | HESI-II | Thermo Fisher Scientific |

**Chromatographic column**

| Types of | specification | manufacturer |
| --- | --- | --- |
| ThermoFisher Hypersil GOLD aQ | 100*2.1 mm, 1.9 μm | Thermo Fisher Scientific |

**Ion source parameter settings**

| Ion source parameters | Set value |
| --- | --- |
| Spray Voltage | 3.5 kV (+)/3.2 kV (-) |
| Capillary Temperature | 320°C |
| Sheath Gas | 35 arb |
| AUX Gas | 10 arb |
| Sweep Gas | 0 arb |
| Probe Heater Temperature | 350°C |
| S-Lens | 60 |

**Gradient elution table**

| Time (min) | Flow (mL/min) | C（有机相）乙腈 | B（水相）0.1%甲酸水 |
| --- | --- | --- | --- |
| 0 | 0.3 | 5 | 95 |
| 2 | 0.3 | 5 | 95 |
| 42 | 0.3 | 95 | 5 |
| 47 | 0.3 | 95 | 5 |
| 47.1 | 0.3 | 5 | 95 |
| 50 | 0.3 | 5 | 95 |
| 柱温：40°C | | | |

**MS scan parameter settings**

| MS scan parameters | Set value |
| --- | --- |
| Scan mode | Full MS-ddms^2^ |
| Full MS scan range | 100 to 1500*m*/*z* |
| Spectrum data type | Profile |
| Resolution | Full MS: 70,000 |
|  | MS/MS: 17,500 |
| AGC target | Full MS:1e6 |
|  | MS/MS:2e5 |
| Maximum IT | Full MS: 100 ms |
|  | MS/MS: 50 ms |
| Loop count | 3 |
| MSX count | 1 |
| Isolation width | 1.5*m*/*z* |
| NCE (Stepped NCE) | 20, 40, 60 |
| Minimum AGC target | 8e3 |
| Intensity Threshold | 1.6e5 |
| Dynamic exclution | 5 s |
